# A Study of the Antimicrobial Activity of Combined Black Pepper and Cinnamon Essential Oils against *Escherichia fergusonii* in Traditional African Yoghurt

**DOI:** 10.3390/foods10112847

**Published:** 2021-11-18

**Authors:** Betty A. Ogwaro, Elizabeth A. O’Gara, David J. Hill, Hazel Gibson

**Affiliations:** 1 Faculty of Science and Engineering, Wolverhampton School of Sciences, University of Wolverhampton, Wulfruna Street, Wolverhampton WV1 1LY, UK; B.A.Ogwaro@wlv.ac.uk (B.A.O.); D.Hill@wlv.ac.uk (D.J.H.); 2 Faculty of Science and Engineering, Research Institute for Healthcare Science, University of Wolverhampton, Wulfruna Street, Wolverhampton WV1 1LY, UK; E.OGara@wlv.ac.uk; 3 Faculty of Science and Engineering, School of Medicine and Clinical Practice, University of Wolverhampton, Wufruna Street, Wolverhampton WV1 1LY, UK

**Keywords:** natural antimicrobial, black pepper extract, cinnamon extract, *Escherichia fergusonii*, traditional yoghurt

## Abstract

The antimicrobial activity of the essential oils of black pepper (BPE) and cinnamon bark (CE) extracts against *E. fergusonii* was assessed in pasteurized full cream milk during and post-fermentation. The milk was fermented with 1% (*v*/*v*) of *Lactobacillus delbrueckii* subspecies *bulgaricus* (NCIMB 11778) and *Streptococcus thermophilus* (NCIMB 10387) (approx. 10^6^ cfu/mL each) and incubated and stored at 25 °C for 5 days (144 h) or at 43 °C for 24 h and then stored at 25 °C for 120 h. The milk was spiked with *E. fergusonii* at the start of fermentation by the lactic acid bacteria (pre-fermentation contamination) for after fermentation (post fermentation contamination). BPE and CE were applied at concentrations based on their minimum inhibitory concentration of 0.5% and 0.25% respectively as follows: 0.5% BPE alone; 0.125% BPE with 0.1875% CE; 0.25% BPE with 0.125% CE; 0.375% BPE with 0.0625% CE; 0.25% CE alone. Results showed that during fermentation at 25 °C, *E. fergusonii* grew to a similar level (approx. 10^9^ CFU/mL) in control samples and 10^8^ CFU/mL when BPE or CE were added alone. Whereas, in the samples with the combined essential oils, the bacterium grew to 10^6^–10^7^ CFU/mL only. During the milk fermentation at 43 °C, *E. fergusonii* grew to approx. 10^9^ CFU/mL in samples without treatment. However, it was not detected in samples containing mixed BPE with CE after 8, 10 and 12 h of fermentation. Subsequent storage at 25 °C resulted in undetectable levels of the bacterium in all the samples treated with BPE or CE after 24 h of storage. These results indicated that BPE in combination with CE reduced growth during fermentation and was bactericidal during storage.

## 1. Introduction

*E. fergusonii* is a Gram-negative, rod-shaped bacterium which is an infrequent but emerging multidrug-resistant human pathogen. It was reported by Farmer et al. [1] in 1985 as a new species of the genus *Escherichia,* family Enterobacteriaceae. It was formerly known as Enteric Group 10 due to its biochemically distinct nature compared to other species and bio-groups of Enterobacteriaceae. It is most closely related to *Escherichia coli* and *Shigella* sp. and more distantly related to other bacterial species of the family Enterobacteriaceae [2]. *E. fergusonii* is one of the five members of *Escherichia* which can be found in the intestines of human beings and can be pathogenic to animals and humans [3,4]. *E. fergusonii* has been isolated from human clinical samples collected from urine, blood, abdominal wounds, faeces and from gall bladder fluids of patients [5]. The bacterium was reported in calves and sheep with clinical cases suggestive of salmonellosis [6]. In horses, goats and ostriches, *E. fergusonii* was associated with symptoms of fibrinonecrotic typhlitis [7,8]. The prevalence of *E. fergusonii* in Africa is not yet well studied. Saad et al. [9] reported the presence of *E. fergusonii* in raw milk and some dairy products in Egypt. In 2017, Glover [10] isolated *E. fergusonii* from non-human primates in South Africa. Adesina [11] identified *E. fergusonnii* from non-clinical samples from patients in a general hospital in Lagos, Nigeria. Similarly, to other *Escherichia* spp., *E. fergusonii* grows optimally on routine culture media at 37–40 °C under aerobic conditions but its temperature range of growth extends up to 45 °C [12,13] and according to Ingle et al. [14] *E. fergusonii* cannot replicate at temperatures below 11 °C.

Since 1985, there has been an increasing number of isolations of *E. fergusonii* in samples from veterinary and human origin, but limited information is known about its growth and survival in foods. The traditional African herders store their excess milk in the form of fermented products such as yoghurt, acidified milk, ghee or cheese to increase its shelf life [15,16]. The traditional farmers add herbs and spices to the milk for flavour and aroma [17]. A large proportion of these traditional products are prepared with raw milk [18] often with poor hygiene and sanitation facilities [19]. Today, herbs and spices form an integral part of foods for flavour and aroma, as well as preservatives and therapeutic agents. They are also used for medicinal preparations, cosmetics, perfumery, and various other products [20,21]. In addition, the WHO [22] noted that between 65% and 80% of the populations of developing countries use medicinal plants as remedies. Furthermore, there is considerable interest in the possible use of natural functional plants as alternative food additives either to prevent the growth of food pathogens or delay the onset of food spoilage [23,24,25]. Essential oils (EOs) from aromatic plants have a high content of bioactive compounds and have widely been used for their bactericidal effects in medical and pharmaceutical fields [26].

Black pepper (*Piper nigrum*) and cinnamon (*Cinnamomum zeylanicum*) are among the spices used in traditional African fermented foods and dairy products [27]. Black pepper, a member of the family Piperaceae, is the most famous spice for flavouring foods throughout the world due to the pungency of its major bioactive component, piperine [26]. Black pepper also contains a wide range of phytochemicals [28]. Besides piperine, it has monoterpenes (camphene, pinene, limonene), other alkaloids [piperinigramides, A-G; piperic ester, pipernigrester A] and flavonoids, which have antimicrobial activities against a range of microorganisms [29]. Moreover, black pepper contains 8% moisture, 10% protein, 10.2% lipid, 66.5% carbohydrate, 4.6% ash and vitamins [30]. Since olden times, *Piper nigrum* has been used aroma and flavour, as well as for medicinal purposes in many parts of the world [31]. Although Nagavekar and Singhal [32] noted that the antimicrobial activities of *Piper nigrum* are due to the presence of oleoresins, in general, the antimicrobial activity of black pepper is primarily due to piperine [33].

Cinnamon (*C. zeylanicum*) on the other hand is from the *Lauraceae* family. It is a common spice in the preparation of tea and beverages in the world. It contains 9.5–10.5% moisture, 3.89–4.65% protein, 59.55–80.59% carbohydrate, 53.1% dietary fibre, 3.55% ash and vitamins [34]. Cinnamaldehyde is the major component of cinnamon bark and cinnamon sticks comprising between 60–90% of the component [35]. Cinnamaldehyde has also been reported to be active as an antibacterial, anti-allergic, anti-ulcerogenic, antipyretic and antioxidant [36,37,38,39]. However, cinnamaldehyde does not affect the growth of *Lactobacillus* species in yoghurt [40]. Traditional herders incorporate cinnamon in fermenting milk for its flavour and aroma. *E. fergusonii* is an infrequent but emerging multidrug-resistant human pathogen and there are reports in the literature that *E. fergusonii* exhibits acquired resistance to a spectrum of antibiotics [41,42,43,44]. With a lack of refrigeration facilities, the traditional farmers ferment excess milk to produce yoghurt and extend the shelf life. However, fermentation acids alone are not enough to control microbial growth.

This study aimed to assess the antimicrobial activity of BPE and/or CE against *E. fergusonii* in full cream milk during or post-fermentation by lactic acid bacteria. The milk was incubated at 25 °C mimicking the traditional African yoghurt fermentation temperature or 43 °C mimicking the industrial temperature.

## 2. Materials and Methods

### 2.1. Media for Growth

Tryptone Soy Broth (TSB, LAB004) Tryptone Soy Agar (TSA, LAB011); de Man-Rogosa-Sharpe Agar (MRSA LAB098); de Man Rogosa-Sharpe Broth (MRSB LAB093) and M17 Agar (LAB092) were all purchased from Lab M Limited, Bury, UK, and M17 Broth (CM0817) was purchased from Thermo Fisher Scientific (Loughborough, UK); All media were prepared according to the manufacturers’ instructions. M17 Agar and M17 broth were used for *S. salivirus* subspecies *thermophilus* [45]. 50 mL of 10% sterilized lactose was added to 1 litre of M17A or M17B before use (46). The sample was tested by streaking on the agar or inoculating in the broth using a typical *S. thermophilus* colony and incubated at 37 °C for 24 h [46]. MRSA and MRSB were used for the enumeration of *L. delbrueckii* subspecies *bulgaricus* [47], and Violet Red Bile Agar (VRBA, CM0107B, Thermo Fischer Scientific, Loughborough, UK) for the enumeration of *E. fergusonii.* Sterile quarter-strength Ringers Solution (BR 0052, Thermo Fischer Scientific, Loughborough, UK) was used as an isotonic diluent for the bacterial cells. All media were prepared with deionized water. The commercial MRS agar had Tween 80 (1.08 g/L) included in the composition of the agar. Thus, no additional components were added to MRSB or MRSA media.

### 2.2. Microorganisms and Culture Conditions

*Escherichia fergusonii* (UCC 585) was an isolate from a traditional African yoghurt, *L. delbrueckii* subspecies *bulgaricus* (NCIMB 11778) and *S. thermophilus* (NCIMB 103687) were obtained from the National Collection of Industrial Food and Marine Bacteria (Aberdeen, UK). Before each experiment, culture from a freeze-dried vial, maintained at −20 °C was activated in TSB for *E. fergusonii*; and in M17 Broth [pH 6.4 ± 0.2] for *S. thermophilus.* Cultures were incubated for 24 h at 37 °C ± 0.1 °C and streaked onto TSA and M17 Agar, respectively. *L. delbrueckii* subspecies *bulgaricus* was activated in MRSB, pH 5.5 ± 0.2 and incubated under anaerobic conditions at 43 ± 0.1 °C for 48–72 h. The cultures were then streaked on MRSA (pH 5.5 ± 0.2) and incubated in an anaerobic gas jar at 43 ± 0.1 °C for 48–72 h. Resuscitated microorganisms were sub-cultured twice before use in the experiments. All activated cultures were maintained on slants at 4 °C and were sub-cultured monthly. The pH of the media was adjusted carefully with 1N HCL.

To prepare the inoculum, one pure isolated colony from an agar plate was transferred to TSB for *E. fergusonii*, M17B [pH 6.4 ± 0.2] for *S. thermophilus* and MRSB [pH 5.5 ± 0.2] for *L. delbrueckii* subspecies *bulgaricus*. *L. delbrueckii* subspecies *bulgaricus* was incubated in an anaerobic gas jar (either 1-litre or 2.5-litre gar jar with a corresponding gas pack). All the broth cultures were incubated at 37 °C for 24 h with shaking (150 rpm). A preliminary assessment was carried out to determine growth and survival of the starter cultures in pasteurized full cream milk with various inoculum sizes and it was found that approx. 10^6^ CFU/mL produced a yoghurt with appropriate coagulation and aroma. It was also determined that both black pepper and cinnamon extract did not affect the growth of the two starter cultures during fermentation. To obtain the viable counts for *E. fergusonii* during the antimicrobial challenge test, serial dilutions were made in ¼ strength Ringer’s solution and 100 µL was plated out on VBRA; incubated for 24 h at 37 °C. Viable counts were recorded after the incubation period.

### 2.3. Pre-Oil Extraction Preparation

Black pepper seeds and cinnamon bark were purchased from a local market in Juba (South Sudan). The spices were first washed with sterile distilled water then dried in a drying cabinet at 50 °C for 72 h. The dried spices were then crushed first individually using a sterilized mortar and then ground with an electric grinder to coarse smaller particles before extraction.

### 2.4. Extraction of Essential Oils

The essential oils (EOs) of black pepper and cinnamon bark were extracted using the Soxhlet extractor according to the methods of Soxhlet [48]. In the first step, 100 g of ground powder of each spice was individually weighed on a calibrated analytical balance directly in a 50 mL porous extraction thimble made from thick filter paper, which was then placed inside the main chamber of the Soxhlet extractor. 500 mL of HPLC grade methanol (Sigma Aldrich, Gillingham, UK) was put in a 1000 mL round bottom flask which was then placed on an electric heater. The heater was turned on to methanol’s boiling point (50 °C). Continuous extraction took place by refluxing the solvent until the extraction was completed. The extraction was left to continue until the solvent was observed to be completely colourless and was left to reflux for a further 2–3 refluxes to ensure complete extraction.

The methanol was evaporated off using a rotary evaporator at a fixed temperature of 50 °C until all the methanol was completely evaporated, leaving a thick essential oil. The samples were kept at −20 °C until use (used within one month). The composition of EOs from the spices was determined in University’s laboratory by Gas Chromatography-Mass spectrometry (data not presented). The main compounds in the cinnamon bark EO were cinnamaldehyde (83.5%) and eugenol (1.02%) and in black pepper seeds were piperine (63.9%) and caryophyllene (6.5%).

### 2.5. Milk Sample Preparation

Whole, full cream pasteurized milk (pH 6.7 ± 0.1) with 3.5% fat was used in this study. The milk was purchased from a supermarket one day before the experiment was performed and at least 6 to 7 days before its use-by date. Sterile test tubes containing 10 mL of milk were heated by steam for 30 min at 85 °C and transferred immediately to cool in a water bath set at 40 °C. The sterility of the samples was confirmed by streaking onto Petri dishes of TSA and incubating at 37 °C for 24 h. The milk samples were used to prepare the different concentrations of antimicrobials in milk.

### 2.6. Determination of Minimum Inhibitory Concentration (MIC)

First, a stock solution of 1% (*v*/*v*) of each EO extract was prepared in methanol (solvent). To determine the minimum inhibitory concentration of the EOs, 12 test tubes each containing 5.0 mL of pasteurized milk were set up. From the 1.0% of spice extract, a 2-fold serial dilution was carried out in tubes 1–10 to give 1 to 0.156%. The 11th and 12th tubes contained pasteurized milk only. 100 µL of a diluted overnight culture of *E. fergusonii* was added to test tubes 1–11 giving a final concentration of approx. 10^5^ CFU/mL in each tube. Tube 11 was *E. fergusonii* and milk only and tube 12 contained pasteurized milk only. All the test tubes were incubated at 37 °C for 24 h. Following incubation, the samples were serially diluted (1:10) in quarter strength Ringer’s solutions and appropriate dilutions were plated on TSA plates. The plates were incubated at 37 °C for 24 h. The lowest concentration of the EO treatment that inhibited visible growth of the pathogen after incubation was taken as the MIC of the treatment. Triplicate samples were included for each treatment and the experiment was replicated 3 times.

### 2.7. Antimicrobial Activity of Combined Essential Oils of Black Pepper Extract (BPE) and Cinnamon Extract (CE) against E. fergusonii

The BPE and CE were used alone at the MIC and in combination at ¼, ½ and ¾ MIC to determine the antimicrobial effectiveness against *E. fergusonii* during fermentation (Table 1). Milk samples were inoculated with 1% each of a mixed culture (*v*/*v*) of *L. delbrueckii* subspecies *bulgaricus* and *S*. *thermophilus* (LAB). 100 µL of a diluted overnight culture of *E. fergusonni* was added to all the 6 tubes to give a final concentration of approx. 10^5^ CFU/mL. Sets of the test tubes were then incubated for 24 h at 25 °C or 43 °C. Growth control tubes (Tube 1) with inoculum but without the spice extracts were included in each experiment. Viable counts during growth were enumerated at 0, 2, 4, 6, 8, 10, 12 and 24 h. The samples were subsequently stored at 25 °C for a further 120 h and survival of the bacterium during storage was evaluated every 24 h until termination of storage. Each set of treatment and test was carried out in triplicate.

For the post-contamination study, a set of samples was inoculated with 1% each of mixed culture (*v*/*v*) culture of *L. delbrueckii* subspecies *bulgaricus* and *S*. *thermophilus* (LAB) only. The sets were incubated at 25 °C or 43 °C for 24 h. Post-fermentation, 2–3 × 10^5^ CFU/mL stationary phase of *E. fergusonii* was added to each sample. The contaminated samples were then treated with combined BPE with CE shown in Table 1. Each treatment was stored at 25 °C for 120 h. Viable counts for *E. fergusonii* were determined by decimal serial dilution in quarter strength Ringers and plating 100 µL on VRBA at 0, 24, 48, 72, 120, 144 h during storage.

### 2.8. pH Measurement

The pH of the samples was measured with a Mettler Toledo Delta 320 pH meter, at room temperature. Readings were taken before inoculation (negative control), immediately after inoculation (T = 0) and then at every sampling point.

### 2.9. Statistical Analysis

All experiments were performed in triplicate. The data were expressed as the mean ± SD. One way ANOVA was used to analyse all the treatments of BPE and/or CE and the control. When the results were significant, two-way ANOVA was used to analyse the interactions between treatments and time. Prism Graph Pad (USA), version 9.0 was used for the statistical analysis.

## 3. Results

### 3.1. Effect of Combined BPE and CE on Growth of E. fergusonii and Change in pH of Milk Fermented at 25 °C

**Figure 1:**
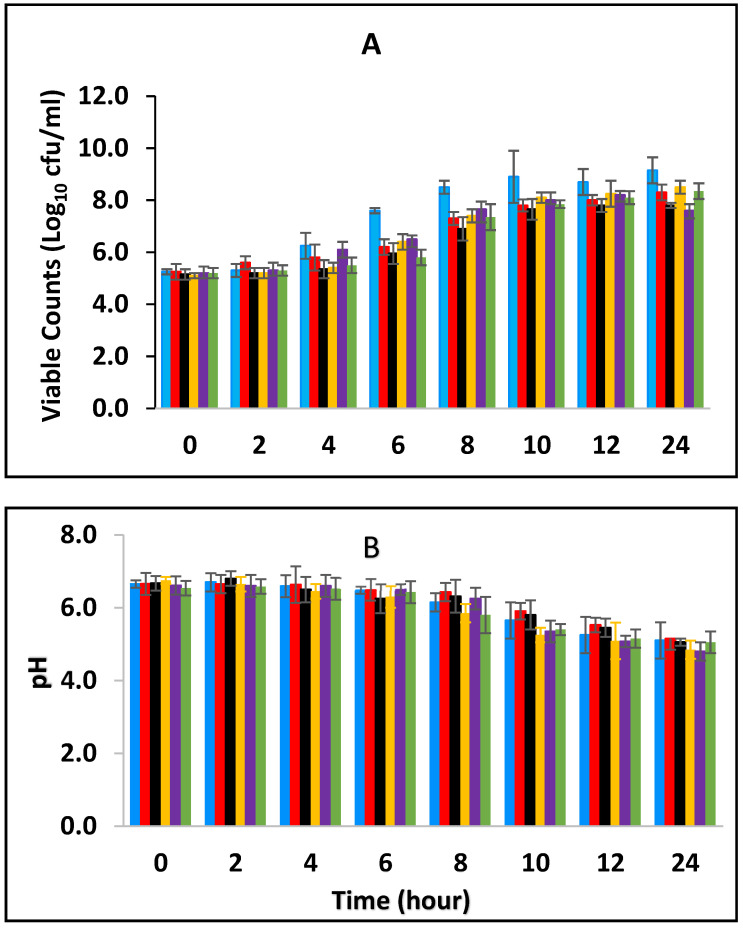
Growth of *E. fergusonii* (**A**) and change in pH (**B**) in fermenting milk in the presence of essential oils of black pepper (BPE) and cinnamon extracts (CE) at different concentrations at 25 °C for 24 h. Concentrations are in % (*w*/*v*), values are the mean of three individual replicates (means ± SD). Control [■]; 0.5% BPE alone [■]; 0.1875% CE + 0.125% BPE [■]; 0.125% CE + 0.25% BPE [■]; 0.0625% CE + 0.375% BPE [■]; 0.25% CE alone [■].

Black pepper and cinnamon extracts were assessed singly and in combination with each other in fermenting/fermented full cream pasteurized milk to determine their effect on *E. fergusonii.* Figure 1A shows the growth curves of *E. fergusonii* incubated at 25 °C in the presence of various concentrations of BPE incorporated together with CE. The result shows that *E. fergusonii* grew in the control sample and it increased from the initial 10^5^ CFU/mL to 10^9^ CFU/mL during fermentation by the lactic acid bacteria at 25 °C (Figure 1A). The counts in the samples treated with the spice extracts varied according to the concentrations but all treatments resulted in significantly lower counts than the control (*p* < 0.05). The most potent combination was 0.375% BPE (three-quarters MIC) combined with 0.0625% CE (one-quarter MIC) with the viable cell counts of approx. 10^7^ CFU/mL, 2 log units lower than the counts in the control samples. In the samples with either BPE or CE alone, the bacterium grew to approx. 10^8^ CFU/mL.

The viable cell counts of lactic acid bacteria were determined at both fermentation temperatures. *S. thermophilus* increased in all the samples from 10^6^ CFU/mL to approximately 10^8^ CFU/mL and 10^9^ CFU/mL at 25 °C and 43 °C respectively and remained consistent until the end of fermentation. *L. delbrueckii* subspecies *bulgaricus* increased to 10^9^ CFU/mL at 24 h at both temperatures showing no inhibition of both *S. thermophilus* and *L. delbrueckii* subspecies *bulgaricus* in the presence of the EOs (Figures S1–S12).

The initial pH of the pasteurized milk was 6.8 (±0.1) but all the samples showed an immediate slight reduction in pH in the milk after incorporation of the EO (Figure 1B). During the fermentation, the pH declined from the initial 6.8 (±0.1) to approximate pH 5.3–5.8 (±0.2) after 10 h of incubation and thereafter to 5.0–5.5 (±0.2) in all the samples. The highest pH reduction was in the sample containing a combination of 0.0625% CE with 0.375% BPE while the least pH reduction was observed at 0.5% BPE (alone). Reduction in pH was rapid in all the samples between 6–10 h of fermentation, which corresponded to the decrease in the growth rate of the pathogen.

### 3.2. Effect of Various Concentrations of BPE and CE on the Growth of E. fergusonii and Change in pH of Milk Fermented at 43 °C

**Figure 2:**
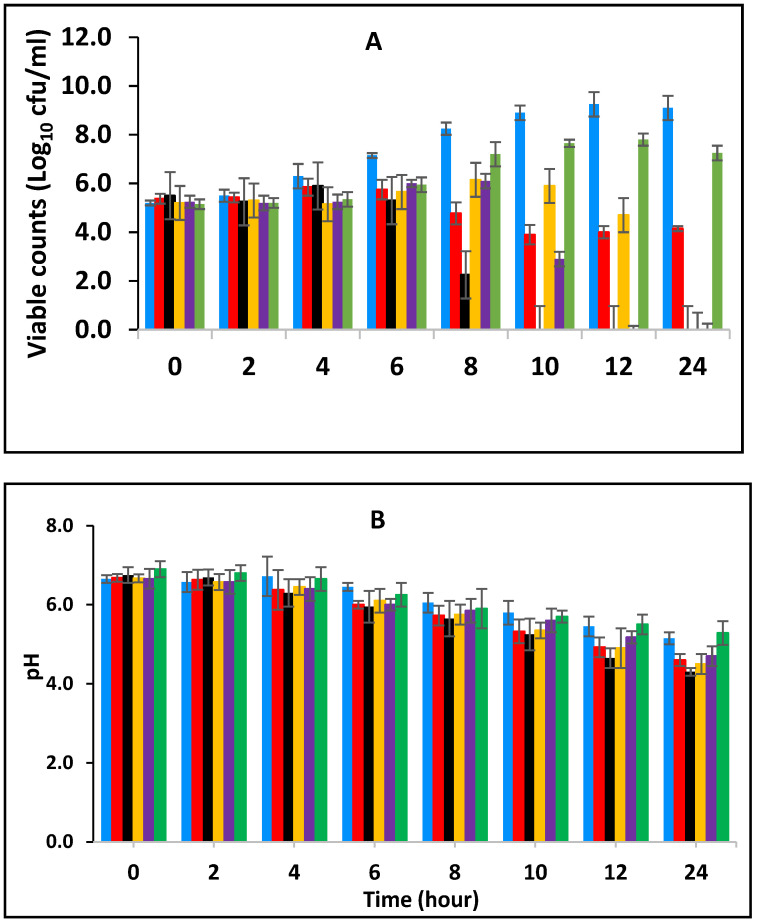
Growth of *E. fergusonii* (**A**) and change in pH (**B**) in fermenting milk in the presence of essential oils of black pepper (BPE) and cinnamon extracts (CE) at different concentrations at 43 °C for 24 h. Concentrations are in % (*w*/*v*), values are the mean of three individual replicates (means ± SD). Control [■]; 0.5% BPE alone [■]; 0.1875% CE + 0.125% BPE [■]; 0.125% CE + 0.25% BPE [■]; 0.0625% CE + 0.375% BPE [■]; 0.25% CE alone [■].

Figure 2 shows the effect of BPE when incorporated together with CE on the growth of *E. fergusonii* and changes in the pH of full cream milk fermented at 43 °C. The results show that *E. fergusonii* counts increased in the control sample to approx. 10^10^ CFU/mL after 12 h of fermentation then declined slightly at 24 h (Figure 2A). Statistical analysis showed a highly significant (*p* < 0.001) difference between the EO treatments after 8 h and fermentation time also had a significant effect (*p* < 0.05). The most effective combination was 0.125% BPE (one-quarter MIC) with 0.1875% CE (three-quarter MIC) and the bacterium was not detected after 8 h of incubation. After ten and twelve hours of fermentation, the bacterium was reduced to undetectable levels in the samples with a combination of 0.25% BPE (one-half MIC) with 0.125% CE (one-half MIC); and 0.375% BPE (three-quarter MIC) with 0.25% CE (one-quarter MIC) respectively. With 0.25% CE, growth of approximately 2 log units was observed in the initial 8 h of fermentation and then growth halted. The presence of 0.5% BPE resulted in an increase of approximately 3 log cycles in the same period of fermentation and reached 1 log unit less than the control sample at 24 h of fermentation.

The pH was lowest in the samples without the addition of spices (pH 4.3–4.8 ± 0.2), while the highest pH was observed in milk containing 0.25% CE alone of pH 5.2 ± 0.2 after 24 h (Figure 2B). In general, the pH declined from the initial 6.8 (±0.1) to average of pH 5.8 (±0.2) after 10 h of incubation and then to 5.0 at 24 h.

### 3.3. Survival of E. fergusonii in Fermented Milk during Storage at 25 °C in Samples Treated with BPE and CE

#### 3.3.1. Survival of *E. fergusonii* during Storage in Milk Fermented at 25 °C

**Figure 3:**
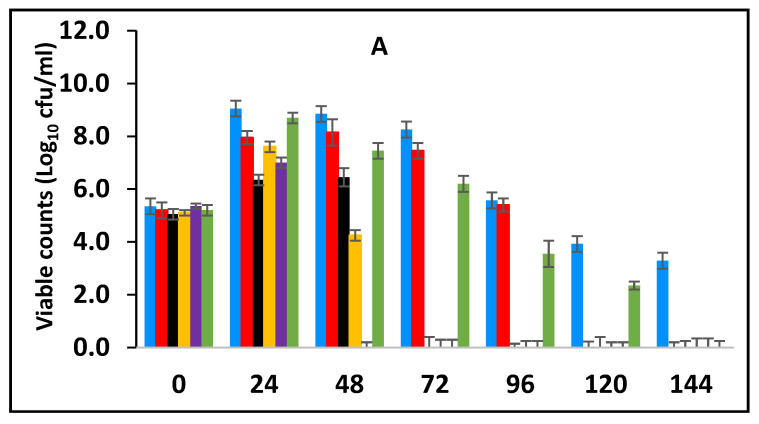

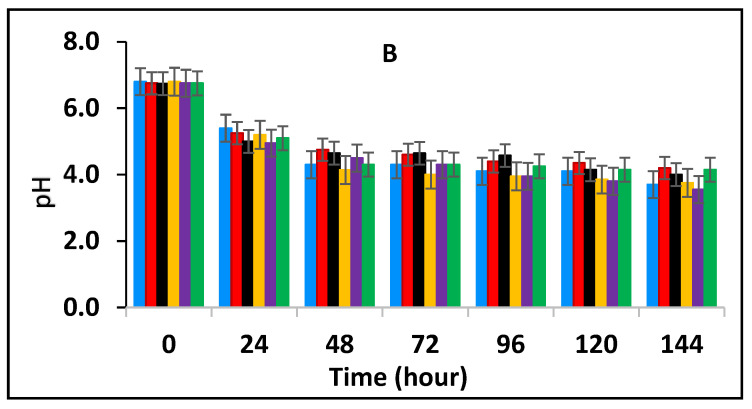
Survival of *E. fergusonii* (**A**) and change in pH (**B**) during storage at 25 °C of milk fermented at 25 °C, in the presence of essential oils of black pepper (BPE) and cinnamon extracts (CE) at different concentrations for 24 h. Concentrations are in % (*w*/*v*), values are the mean of three individual replicates (means ± SD). Control [■]; 0.5% BPE alone [■]; 0.1875% CE + 0.125% BPE [■]; 0.125% CE + 0.25% BPE [■]; 0.0625% CE + 0.375% BPE [■]; 0.25% CE alone [■].

In this section, the effect of subsequent storage at 25 °C on the survival of *E. fergusonii* in the milk fermented at 25 °C in the presence of various concentrations of BPE and CE was determined (Figure 3A). Traditionally farmers add some spices during milk fermentation mainly for their aroma and flavour but also possibly to control the growth of any pathogens in the milk. Milk is fermented in the warmer part of the house (25–30 °C) and after fermentation, the farmer stores the fermented milk at room temperature (20–30 °C) for a period between three to five days before consumption [49]. *L. delbrueckii* subspecies *bulgaricus and S. thermophilus* were incorporated with BPE together with CE and subsequently, the samples were maintained at 25 °C (Figure 3) simulating African room temperatures and practice for both fermentation and storage of the product which effectively extended the fermentation time. Figure 3 shows that the treatments which included both BPE and CE had a highly significant effect (*p* < 0.001) on *E. fergusonii* and the bacterium was not detected after 24 h of storage in the samples containing 0.375% BPE combined with 0.0625% CE. In the samples containing 0.25% BPE combined with 0.125% CE the organism was not detected after 48 h of storage. There was also a significant interaction between treatment and storage time (*p* < 0.001).

At the point of storage, the pH values were between pH 5.0–5.4 (±0.2) (Figure 3B). The pH was approx. 5.0 (±0.2) in samples containing 0.375% BPE combined with 0.0625% CE and pH 5.2 (±0.2) in the control samples. Throughout the storage period, the pH of the samples continued to decline initially sharply, but later less rapidly. At 144 h of storage, the pH in the control samples was approx. pH 4.2 ± 0.2 and the samples with EOs also declined over this period suggesting the continued fermentation of the milk by lactic acid bacteria. However, the pH did not decline to the same extent in samples with a single EO.

#### 3.3.2. Survival of *E. fergusonii* during Storage in Milk Fermented at 43 °C

**Figure 4:**
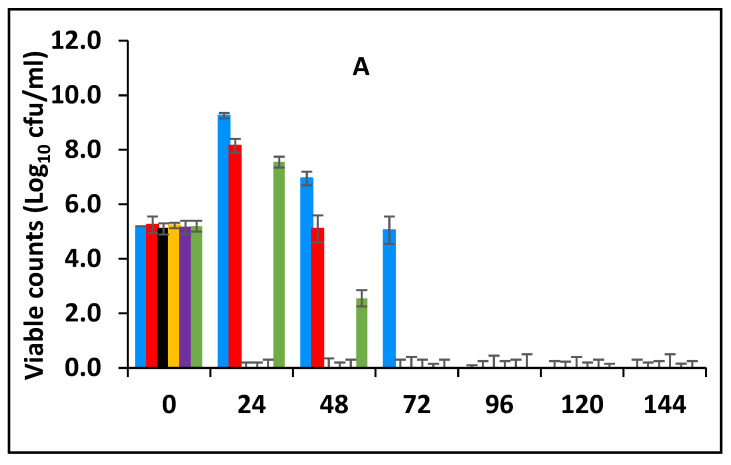

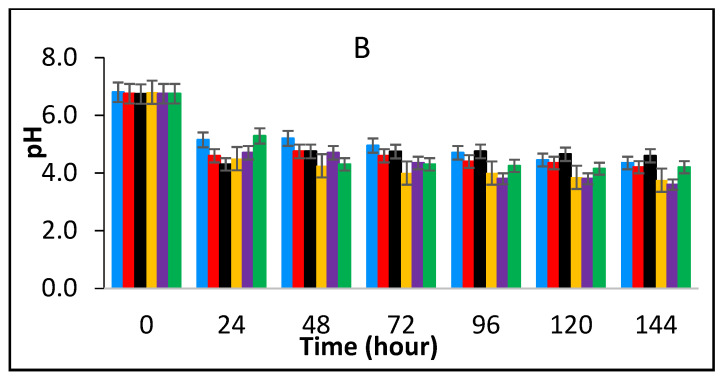
Survival of *E. fergusonii* (**A**) and change in pH (**B**) during storage at 25 °C of milk fermented at 43 °C, in the presence of essential oils of black pepper (BPE) and cinnamon extracts (CE) at different concentrations for 24 h. Concentrations are in % (*w*/*v*), values are the mean of three individual replicates (means ± SD). Control [■]; 0.5% BPE alone [■]; 0.1875% CE + 0.125% BPE [■]; 0.125% CE + 0.25% BPE [■]; 0.0625% CE + 0.375% BPE [■]; 0.25% CE alone [■].

Figure 4A shows the effect of fermentation at 43 °C for 24 h and subsequent storage at 25 °C in the presence of spices, on the survival of *E. fergusonii*. During fermentation at 43 °C prior to storage, the bacterium was undetectable in samples containing, 0.125% BPE with 0.1875% CE; 0.125% BPE with 0.1875% CE; 0.25% BPE with 0.125% CE and 0.375% BPE with 0.0625% CE. After storage at 25 °C for 144 h, the bacterium was not detected in any of the samples. In samples with 0.5% BPE and 0.25% CE, there was a rapid decline in the viable cell counts of the bacterium during storage at 25 °C and it was not detected after 48 h of storage. In all, storage of milk fermented at 43 °C, co-inoculated with *E. fergusonii* resulted in a significant decrease of viable cells to undetectable levels over the 5-day storage period (Figure 4A). The inability of the *E. fergusonii* to maintain its viability correlates with the decline of the pH throughout storage (Figure 4B) from the initial pH 6.8 to between 4.8 (±0.2) for samples containing 0.375% BPE combined with 0.0625% CE and to pH 5.2 (±0.2) in the control samples. After 144 h of storage, the control samples and the samples with 0.25% CE alone had the lowest pH (slightly below pH 4.0 ± 0.2) suggesting the continued fermentation of the milk by lactic acid bacteria.

### 3.4. Post Fermentation Survival of E. fergusonii

#### 3.4.1. Survival of *E. fergusonii* Inoculated into Milk after Fermentation at 25 °C and Subsequently Stored at 25 °C

**Figure 5:**
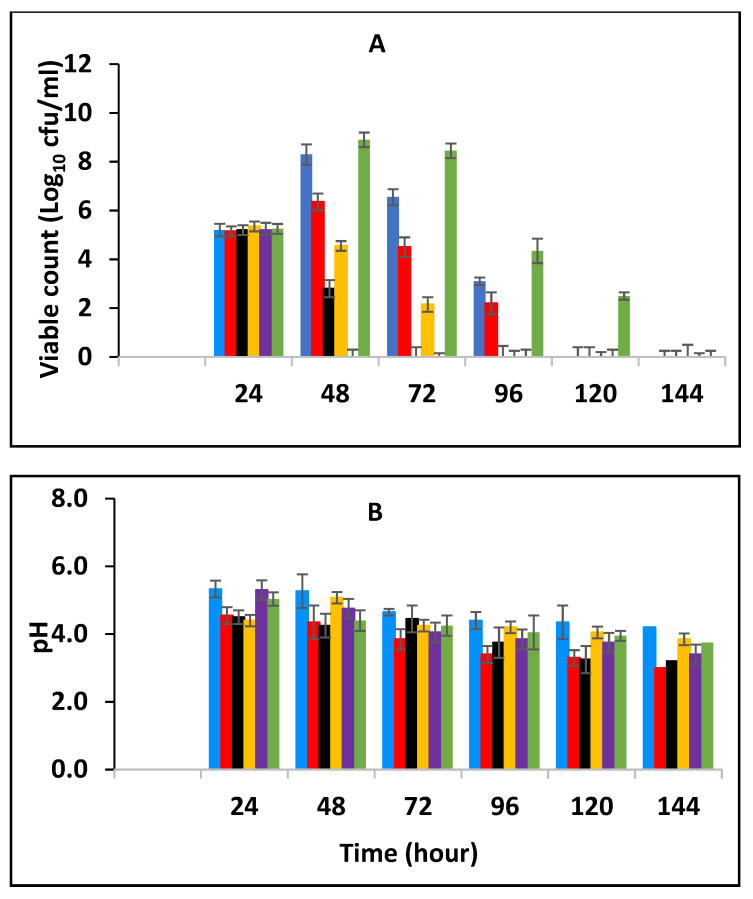
Survival of *E. fergusonii* (**A**) and change in pH (**B**) in the presence of essential oils of black pepper (BPE) and cinnamon extracts (CE) during storage at 25 °C of yoghurt fermented at 25 °C for 24 h. The EOs were added at different concentrations post milk fermentation. Concentrations are in % (*w*/*v*), values are the mean of three individual replicates (means ± SD). Control [■]; 0.5% BPE alone [■]; 0.1875% CE + 0.125% BPE [■]; 0.125% CE + 0.25% BPE [■]; 0.0625% CE + 0.375% BPE [■]; 0.25% CE alone [■].

Milk was fermented at 25 °C and then inoculated with 10^5^ CFU/mL of stationary phase *E. fergusonii* (post-fermentation contamination) in addition to various combinations of BPE and CE of 0.5% BPE alone; 0.125% BPE with 0.1875% CE; 0.25% BPE with 0.125% CE; 0.375% BPE with 0.0625% CE; 0.25% CE alone as natural antimicrobials. A control sample without the spices was also included and all were stored at 25 °C for 5 days (Figure 5A). The samples containing both BPE and CE showed a significant reduction in *E. fergusonii* compared to the control (*p* < 0.05) and this was time-dependent. After 24 h of storage, the viable cell numbers declined from the initial 10^5^ CFU/mL to undetectable levels in the samples containing 0.375% BPE with 0.0625% CE indicating the effect of BPE as it was three-quarters of the combination. The results in Figure 5A show that the combination that was next most effective was 0.125% BPE with 0.1875% CE, as the bacterium was undetectable after 48 h of storage. However, in samples with EO alone (0.5% BPE or 0.25% CE), the bacterium took longer to demise and was not recovered after 3 and 4 days of storage which was similar to the control.

The effect of pH is presented in Figure 5B. The initial pH at the point of storage was 5.1 (±0.2). During the storage period, the pH of the samples declined in all the samples by approximately 1.2–1.5 pH units in all the other samples. The highest reduction was when CE was applied alone. The action of the bacterial enzyme on lactose results in the production of lactic acids and acetaldehyde which reduced the pH and contributed to the demise of *E. fergusonii* during storage. The spices did not affect the growth of LAB significantly but did reduce the rate of the pH decline of the fermenting milk.

#### 3.4.2. Survival of *E. fergusonii* Inoculated into Milk after Fermentation at 43 °C and Subsequently Stored at 25 °C

**Figure 6:**
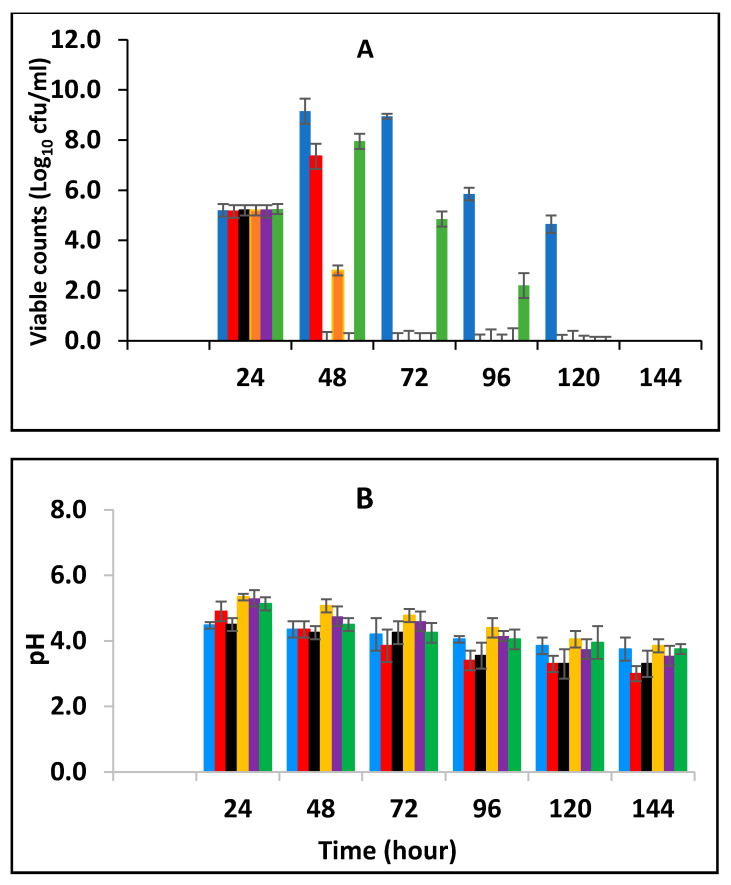
Survival of *E. fergusonii* (**A**) and change in pH (**B**) in the presence of essential oils of black pepper (BPE) and cinnamon extracts (CE) during storage at 25 °C of yoghurt fermented at 43 °C for 24 h., The EOs were added at different concentrations post milk fermentation. Concentrations are in % (*w*/*v*), values are the mean of three individual replicates (means ± SD). Control [■]; 0.5% BPE alone [■]; 0.1875% CE + 0.125% BPE [■]; 0.125% CE + 0.25% BPE [■]; 0.0625% CE + 0.375% BPE [■]; 0.25% CE alone [■].

Milk was fermented at 43 °C and then inoculated with 10^5^ CFU/mL of stationary phase *E. fergusonii* (post-fermentation contamination) in addition to varied concentrations of BPE and CE then subsequently stored at 25 °C. Figure 6 showed that storage at 25 °C resulted in a decrease of viable *E. fergusonii* cells to undetectable levels within 24 h of storage in the samples with the concentrations of 0.375% BPE (three-quarters MIC) combined with 0.0625% CE (one-quarter MIC). Similarly, after 48 h, viable cells were not detected in the samples with concentrations of 0.125% BPE with 0.1875% CE or 0.25% BPE with 0.125%. The viable cell population continued to decline to undetectable levels after 72 h. The effect of pH during storage at 25 °C after fermentation at 43 °C is presented in Figure 6B. The initial pH was pH 5.2 (±0.2) and it declined in all the samples, however, fermentation without treatment with EOs had the lowest pH.

## 4. Discussion

In this study, we observed the effect of BPE combined with CE against *E. fergusonii* in full cream pasteurized milk fermented at 25 °C to simulate ambient, African fermentation temperature, and at 43 °C, the industrial fermentation temperature. The higher fermentation temperature of 43 °C resulted in a more rapid decline in *E. fergusonii* than in milk fermented at 25 °C. The results in Figure 1 and Figure 2 showed that the higher temperatures resulted in a lower pH due to the acid production during the growth of the lactic acid bacteria. It also showed that the EO did not affect acidification by the LAB at the higher temperature. At the lower temperature (25 °C), the pH reduction was less marked due to the slower growth of the LAB. At 43 °C, the bacterium was completely inactivated after twenty-four hours of incubation whereas, at 25 °C, the pathogen was detected in all the treatments, although at counts lower than that of the control samples. A similar effect of fermentation temperature was reported by Visvalingam and Holley [50] on *E. coli*. The degree of cell reduction depended on the combination of concentrations of the EOs (Figure 1 and Figure 2).

The MIC of BPE (0.5%) and CE (0.25%) against *E. fergusonii* are comparable with the value reported by Cava et al., [51] for the MIC of cinnamon extract (0.31%) against *E. coli* in pasteurized milk. In this study, the effect on the viable counts in the samples treated with the spice extracts varied according to the concentrations. The combination of one-quarter fraction of MIC of BPE (0.125%) to three-quarters of MIC of CE (0.1875%) reduced the growth of *E. fergusonii* to a greater extent than the other combinations. The next most potent combination was 0.375% BPE (three-quarters MIC) combined with 0.0625% CE (one-quarter MIC). The results showed that cinnamon extract was enhanced by black pepper extract if the concentration of the cinnamon extract was three times the MIC of BPE. These results suggest that CE and BPE in combination are effective EOs capable of improving the effectiveness of milk fermentation to inhibit *E. fergusonii*.

The results showed that the higher fermentation temperature resulted in a more marked reduction in cell numbers and reflected the reduction in pH of the medium due to lactic acid production. The addition of spices was an added stress on the cells and may have led to more inhibition at the higher temperature of 43 °C. Althair et al. [52] indicated that heat treatment had effects on the physicochemical properties of bacteria during *mudafra* cheese ripening. With emerging drug and acid-resistant microorganisms in recent years, natural antimicrobials that can act synergistically are crucial in controlling foodborne pathogens. Essential oils could serve as promising tools in enhancing food preservation and a similar conclusion was reached by Pavác et al. [53]. In this study, fermentation at African room temperature (25 °C) was not sufficient on its own to inhibit the bacterium as the pH was approx. 5 after 24 h. Similarly, Ogwaro et al., [54] reported a rapid decline in the numbers of *E. coli* O157:H7, another drug-resistant bacterium, only when the pH had reached pH 4.4 and below. In addition, post-fermentation contamination is a concern and this was apparent during the storage of the fermented milk at 25 °C (3–5 days). This work has shown that lactic fermentation in the presence of the EOs enhances the effect of acidification of the fermented milk and reduces the subsequent survival of *E. fergusonii*. The bacterium reached undetectable levels when the pH of the milk was still higher than pH 5.0. This suggests a synergetic reaction between the fermentation acids in combination with the EOs.

This work has shown that the addition of spice essential oils enhances the inhibition of the bacterium. Furthermore, according to Moon et al., [55], at low pH, the antimicrobial molecules bind better to the hydrophobic zone of the membrane, where they are diluted in the lipid phase, improving their activities on bacteria and fungi. In addition, to the temperature of fermentation, the effect of the combination of the spices showed higher antimicrobial activity compared to a single application (1 MIC) of both EOs. These findings may be useful for food applications, but their effect on the sensory quality of various foods needs to be studied. The effect of the combined concentrations had more impact at the higher fermentation temperature (43 °C) than at the lower fermentation temperature (25 °C) as seen in Figure 1 and Figure 2. It is evident from these results that the combinations of the EOs had a greater impact than when the EOs were applied alone, suggesting additive activity. Moreover, where the amount of CE was higher than BPE, the inhibition increased. This is an indication that BPE acted as a facilitator by enhancing the activity of CE. Enhanced antimicrobial activity of EOs at the higher temperature could be due to an increased rate of cellular metabolism, growth and death. The increased inhibition at 43 °C, the higher temperature, could be also due to increased fluidity of the cytoplasmic membrane that occurs at warmer temperatures [56]. The results show that there was a positive association between antimicrobial concentration and microbial inhibition although this effect was variable.

The mode of action of cinnamaldehyde has been shown to be inhibition of amino acid decarboxylases in *Enterobacter aerogenes* [57,58] due to the carbonyl group binding to proteins, thus preventing the action of these decarboxylases. Hyidgaard et al. [59] reported that trans-cinnamaldehyde was capable of inhibiting *E. coli* and *Salmonella* by gaining access to the periplasm and inhibiting the activity of transmembrane ATPase. However, Helander et al. [60] found that trans-cinnamaldehyde was capable of inhibiting *E. coli* without gaining access to the periplasm as well as the deeper parts of the bacterial cell. In research conducted by Gill and Holley [61], increasing the concentration of cinnamaldehyde (0.0136–0.1362%) was found to decrease ATPase activity of isolated cell membranes. This is consistent with the highest impact on the cells being observed at 0.1875% CE, which is higher than that tested by Gill and Holley [62]. The effect of the EOs depended on the composition of the mixture of the two EOs as well as the temperature of fermentation.

After fermentation, the samples were subsequently stored at 25 °C and the effect of storage on *E. fergusonni* was observed. This assessment showed that microbial reduction was promoted by EOs during storage. Exposure time had a strong influence on microbial reduction, but also that such an effect was dependent on when the antimicrobial was added to the milk, either pre-fermentation or post-fermentation. When the antimicrobial was added post-fermentation and then stored, the EOs were more effective than when applied pre-fermentation. This suggests that for pathogen inactivation, and to achieve a certain microbial reduction, distinct exposure times to the EOs are required. This is also related to the pH of the sample. It was also observed that when adding the EOs post milk fermentation a faster inhibitory rate of inhibition occurred compared to adding the EOs to the milk pre-fermentation (Figure 3, Figure 4, Figure 5 and Figure 6).

The effect of subsequent storage at 25 °C on *E. fergusonii* showed that the effect was highest with the concentrations of 0.375% BPE (three-quarters MIC) with 0.0625% CE (one-quarter MIC). This is contrary to the effect observed during fermentation, where the effect of the EOs were stronger at the combinations of the concentrations of 0.125% BPE (one-quarter MIC) with 0.1875% CE (three-quarters MIC) was highest. Patil et al. [63] reported that piperine, the major pungent component of black pepper can enhance the bioavailability of other drugs due to its low aqueous solubility and in fermented milk, where the casein has been broken down, provides better access to the cell membrane. Storage at 25 °C was to simulate the ambient and storage temperature of traditional yoghurt in rural Africa. However, this temperature could also be considered a temperature abuse for storage because enteric bacteria can grow at this temperature which could result in the growth of foodborne pathogens and increase the risk of illness from foodborne diseases. It was observed that increased storage time of the fermented milk at 25 °C resulted in a further reduction in pH of the fermented milk and in combination with BPE and CE limited the survival of the *E. fergusonii.*

Several important variables were observed when EOs were applied in the fermenting full cream milk assay. It was evident that the antimicrobial activities of EOs were affected by the contact time of the oils on the bacterium and the concentration required to achieve the same degree of inhibition. These two variables interacted with each other. The results showed that incorporation of EOs into yoghurt (post-fermentation) was more effective in inhibiting *E. fergusonii*, whereas application in pre-fermentation of milk resulted in a lower inhibitory effect. When BPE was applied in combination with CE to lactic fermenting milk, reduced cell counts were observed during storage rather than during fermentation. This could mean that the effect of pH had already weakened the cells thus adding another stress factor such as the EOs was able to penetrate the cells causing rapid death.

The third factor in the extent of the inactivation of the bacterium was pH. The effect of pH and lactic acid concentration play a crucial role in inhibiting Enterobacteriaceae in yoghurt. At low pH, yeasts and moulds are an issue particularly if fruits are added. The addition of whole unextracted spices by farmers into yoghurts could also pose risks of contamination and spoilage so for this reason spice extract could be a better alternative for farmers. As the pH declined to 5.2 and below, the antimicrobial effects of the EOs became more pronounced. The higher inhibition of combined spice extracts in full cream pasteurized milk at 43 °C rather than at 25 °C can be attributed to the influence of low pH. Fitzgerald et al. [64] reported the effect of pH when they assessed the antimicrobial activity of vanillin in apple juice. The antibacterial activity of cinnamon bark extract may be attributed to the high presence of active compounds like cinnamaldehyde and other minor components such as alkaloids, tannins, terpenes and saponins which may act additively or synergistically in inhibiting bacterial growth, causing cell structural damage and leakage of cellular contents and cinnamaldehyde also interferes with electron transfer in cells. Yossa et al. [65] used scanning and transmission electron microscopy of cinnamon oil-treated bacterial cells and reported that there was cell structural damage and leakage of cellular content.

While it is desirable to add concentrations that can inhibit the bacteria rapidly in fermenting/fermented milk, this will be dependent upon the organoleptic acceptability of cinnamon bark or black pepper as their EOs have strong flavours. The synergistic effects of the two spices with each other and with fermentation acids on *E. fergusonii* should be exploited to optimize the concentration required to achieve the desired antibacterial effect without adversely affecting the organoleptic acceptability of the fermented milk. Thus, the use of combined technology could produce fermented foods of acceptable sensory quality and microbial safety. Furthermore, a good method of encapsulation of the EOs could be a solution to the use of natural antimicrobials of plant origin whilst minimizing organoleptic impact. It could be a practical way to enhance the microbiological safety of this traditional yoghurt produced by rural farmers.

## Figures and Tables

**Table 1:** Concentrations of black pepper extract (BPE) and cinnamon extract (CE) were evaluated for antimicrobial effects against *E. fergusonii*.

Tube No.	BPE conc. (%)	CE conc. (%)	MIC of BPE	MIC of CE
1	0	0	0	0
2	0.5	0	1	0
3	0.125	0.1875	¼	¾
4	0.25	0.125	½	½
5	0.375	0.0625	¾	¼
6	0	0.25	0	1

## Data Availability

The data presented in this study are openly available.

## Supplementary Materials

The following are available online at https://www.mdpi.com/article/10.3390/foods10112847/s1, Figure S1. Growth curves of *L. delbrueckii* subspecies *bulgaricus* in fermenting milk in the presence of essential oils of black pepper (BPE) and cinnamon extracts (CE) at different concentrations at 25°C for 24 h. Concentrations are in % (*w*/*v*), values are the mean of three individual replicates (means ± SD). Figure S2. Growth curves of *L. delbrueckii* subspecies *bulgaricus* in fermenting milk in the presence of essential oils of black pepper (BPE) and cinnamon extracts (CE) at different concentrations at 43 °C for 24 h. Concentrations are in % (*w*/*v*), values are the mean of three individual replicates (means ± SD). Figure S3. Growth of *L. delbureckii* subspecies *bulgaricus* in fermenting milk in the presence of essential oils of black pepper (BPE) and cinnamon extracts (CE) at different concentrations during storage at 25 °C after fermentation at 25 °C for 24 h. Concentrations are in % (*w*/*v*), values are the mean of three individual replicates (means ± SD). Figure S4. Growth of *L. delbrueckii* subspecies *bulgaricus* in fermenting milk in the presence of essential oils of black pepper (BPE) and cinnamon extracts (CE) at different concentrations during storage at 25 °C after fermentation at 43 °C for 24 h. Concentrations are in % (*w*/*v*), values are the mean of three individual replicates (means ± SD). Figure S5. Growth of *L. delbrueckii* subspecies *bulgaricus* during subsequent storage at 25°C of the milk fermented at 25°C for 24 h subsequently incorporated with black pepper extract (BPE) combined with cinnamon extract (CE) at different concentrations. Concentrations are in % (*w*/*v*), values are the mean of three individual replicates (means ± SD). Figure S6. Growth of *L. delbrueckii* subspecies *bulgaricus* during subsequent storage at 25 °C of the milk fermented at 43 °C for 24 h subsequently incorporated with black pepper extract (BPE) combined with cinnamon extract (CE) at different concentrations. Concentrations are in % (*w*/*v*), values are the mean of three individual replicates (means ± SD). Figure S7. Growth of *S. thermophilus* in fermenting milk in the presence of essential oils of black pepper (BPE) and cinnamon extracts (CE) at different concentrations at 25 °C for 24 h. Concentrations are in % (*w*/*v*), values are the mean of three individual replicates (means ± SD). Figure S8. Growth of *S. thermophilus* in fermenting milk in the presence of essential oils of black pepper (BPE) and cinnamon extracts (CE) at different concentrations at 43 °C for 24 h. Concentrations are in % (*w*/*v*), values are the mean of three individual replicates (means ± SD). Figure S9. Growth of *S. thermophilus* in fermenting milk in the presence of essential oils of black pepper (BPE) and cinnamon extracts (CE) at different concentrations fermented at 25 °C for 24 h. Concentrations are in % (*w*/*v*), values are the mean of three individual replicates (means ± SD). Figure S10. Growth of *S. thermophilus* in fermenting milk in the presence of essential oils of black pepper (BPE) and cinnamon extracts (CE) at different concentrations during storage at 25°C after fermentation at 43°C for 24 h. Concentrations are in % (*w*/*v*), values are the mean of three individual replicates (means ± SD). Figure S11. Survival of *S. thermophilus* during subsequent storage at 25°C of the milk fermented at 25 °C for 24 h subsequently incorporated with black pepper extract (BPE) combined with cinnamon extract (CE) at different concentrations. Concentrations are in % (*w*/*v*), values are the mean of three individual replicates (means ± SD). Figure S12. Survival *S. thermophilus* during subsequent storage at 25 °C of the milk fermented at 43 °C for 24 h then incorporated with black pepper (BPE) and cinnamon extracts (CE) at different concentrations. Concentrations are in % (*w*/*v*), the mean of three individual replicates (means ± SD).
