# Supplementary material for: A Study of the Antimicrobial Activity of Combined Black Pepper and Cinnamon Essential Oils against Escherichia fergusonii in Traditional African Yoghurt
Source: Foods. 2021 Nov 18;10(11):2847. doi: 10.3390/foods10112847 (PMC8618451; doi:10.3390/foods10112847)
Supplement: Supplementary file 1 [file foods-10-02847-s001.zip › foods-1443888-supplementary.pdf]

Supplementary Figures

Figures for growth at 25°C or 43°C and survival during storage at 25°C of *L. delbrueckii* subspecies *bulgaricus* (Figures S1-S6) co-cultured with *S. thermophilus* (Figures S7-S12) in milk incubated in the presence of essential oils of black pepper (BPE) and cinnamon extracts (CE) at different concentrations.

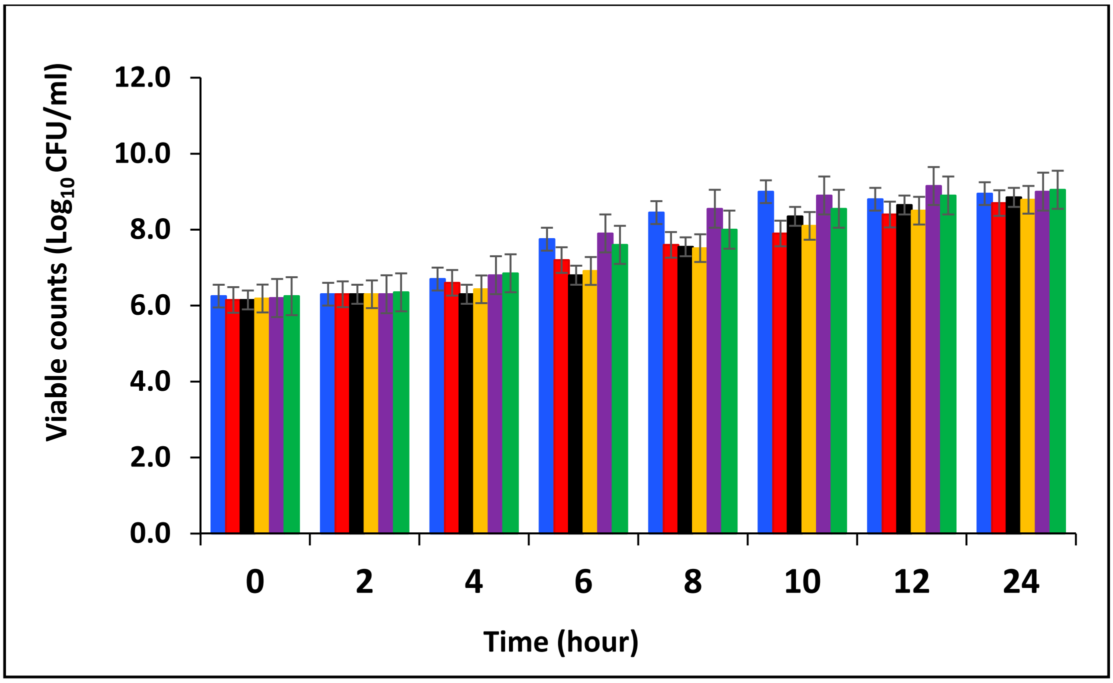

Figure S1. Growth curves of *L. delbrueckii* subspecies *bulgaricus* in fermenting milk in the presence of essential oils of black pepper (BPE) and cinnamon extracts (CE) at different concentrations at 25°C for 24 h. Concentrations are in % (w/v), values are the mean of three individual replicates (means ± SD) Control [■]; 0.5% BPE alone [■]; 0.1875% CE + 0.125% BPE [■]; 0.125% CE + 0.25% BPE [■]; 0.0625% CE + 0.375% BPE [■]; 0.25% CE alone [■].

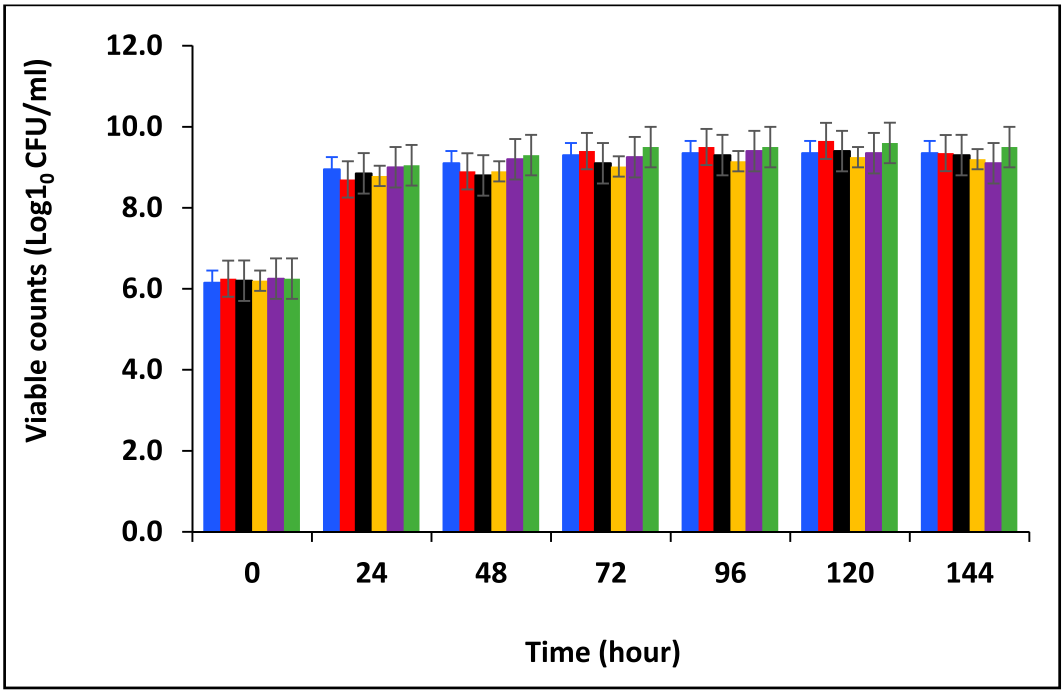

Figure S2. Growth curves of *L. delbrueckii* subspecies *bulgaricus* in fermenting milk in the presence of essential oils of black pepper (BPE) and cinnamon extracts (CE) at different concentrations at 43°C for 24 h. Concentrations are in % (w/v), values are the mean of three individual replicates (means ± SD). Control [■]; 0.5% BPE alone [■]; 0.1875% CE + 0.125% BPE [■]; 0.125% CE + 0.25% BPE [■]; 0.0625% CE + 0.375% BPE [■]; 0.25% CE alone [■].

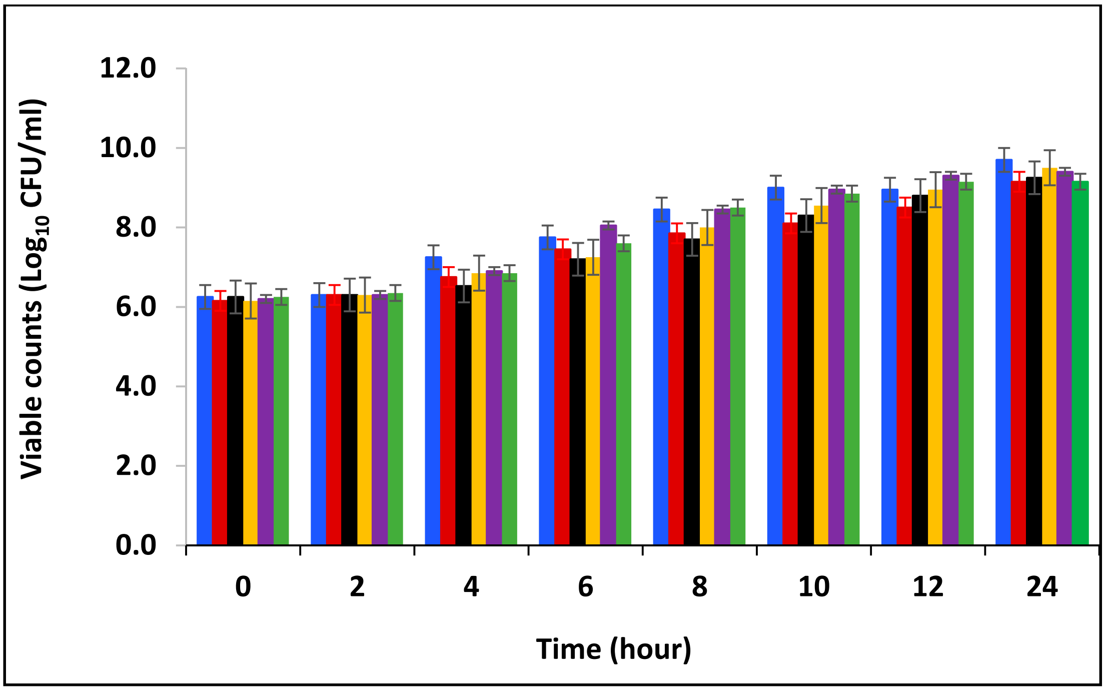

Figure S3. Growth of *L. delbureckii* subspecies *bulgaricus* in fermenting milk in the presence of essential oils of black pepper (BPE) and cinnamon extracts (CE) at different concentrations during storage at 25°C after fermentation at 25°C for 24 h. Concentrations are in % (w/v), values are the mean of three individual replicates (means ± SD). Control [■]; 0.5% BPE alone [■]; 0.1875% CE + 0.125% BPE [■]; 0.125% CE + 0.25% BPE [■]; 0.0625% CE + 0.375% BPE [■]; 0.25% CE alone [■].

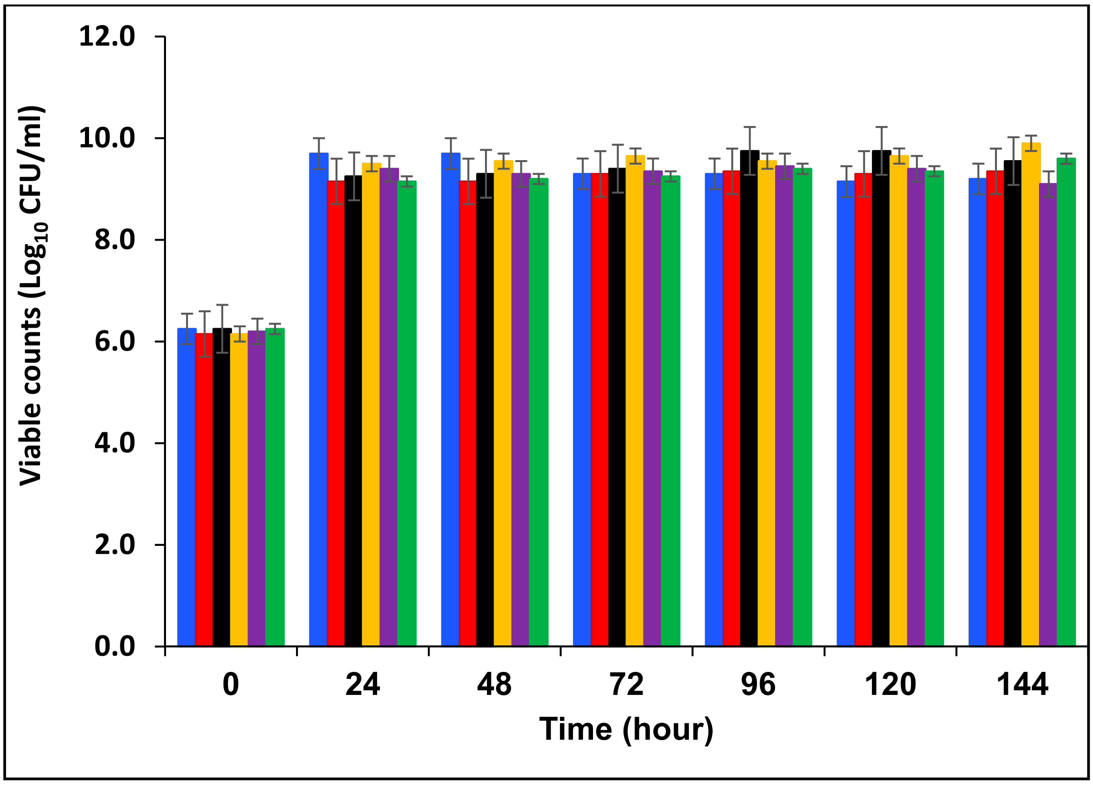

Figure S4. Growth of *L. delbrueckii* subspecies *bulgaricus* in fermenting milk in the presence of essential oils of black pepper (BPE) and cinnamon extracts (CE) at different concentrations during storage at 25°C after fermentation at 43°C for 24 h. Concentrations are in % (w/v), values are the mean of three individual replicates (means ± SD). Control [■]; 0.5% BPE alone [■]; 0.1875% CE + 0.125% BPE [■]; 0.125% CE + 0.25% BPE [■]; 0.0625% CE + 0.375% BPE [■]; 0.25% CE alone [■].

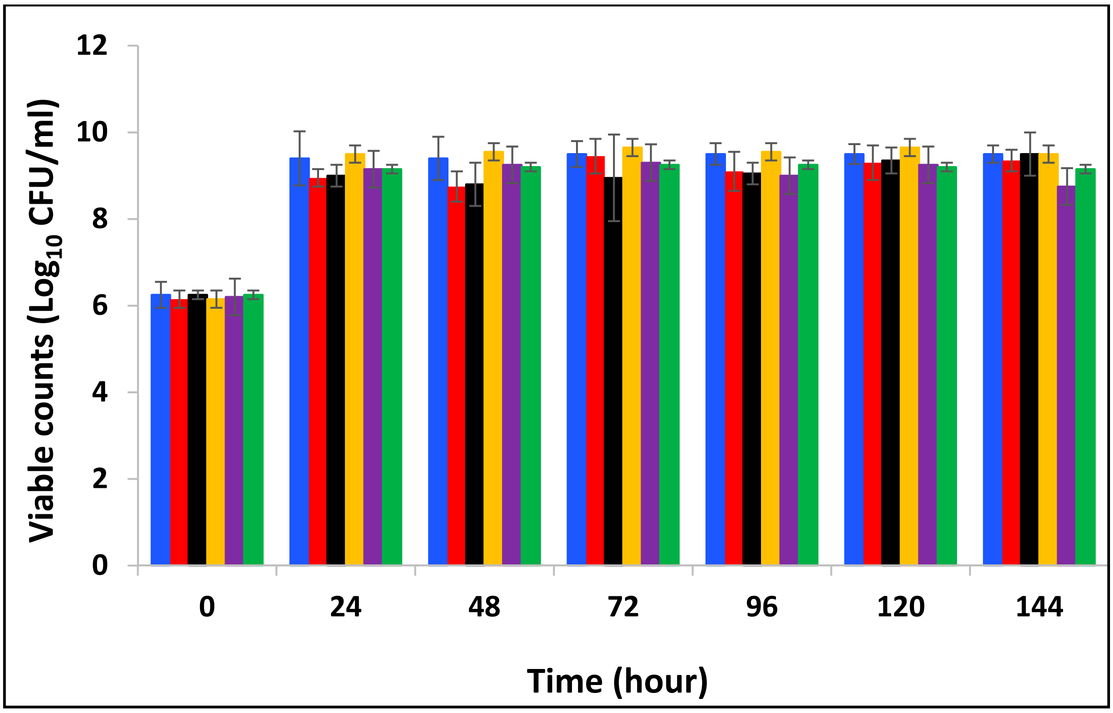

Figure S5. Growth of *L. delbrueckii* subspecies *bulgaricus* during subsequent storage at 25°C of the milk fermented at 25°C for 24 h subsequently incorporated with black pepper extract (BPE) combined with cinnamon extract (CE) at different concentrations. Concentrations are in % (w/v), values are the mean of three individual replicates (means ± SD). Control [■]; 0.5% BPE alone [■]; 0.1875% CE + 0.125% BPE [■]; 0.125% CE + 0.25% BPE [■]; 0.0625% CE + 0.375% BPE [■]; 0.25% CE alone [■].

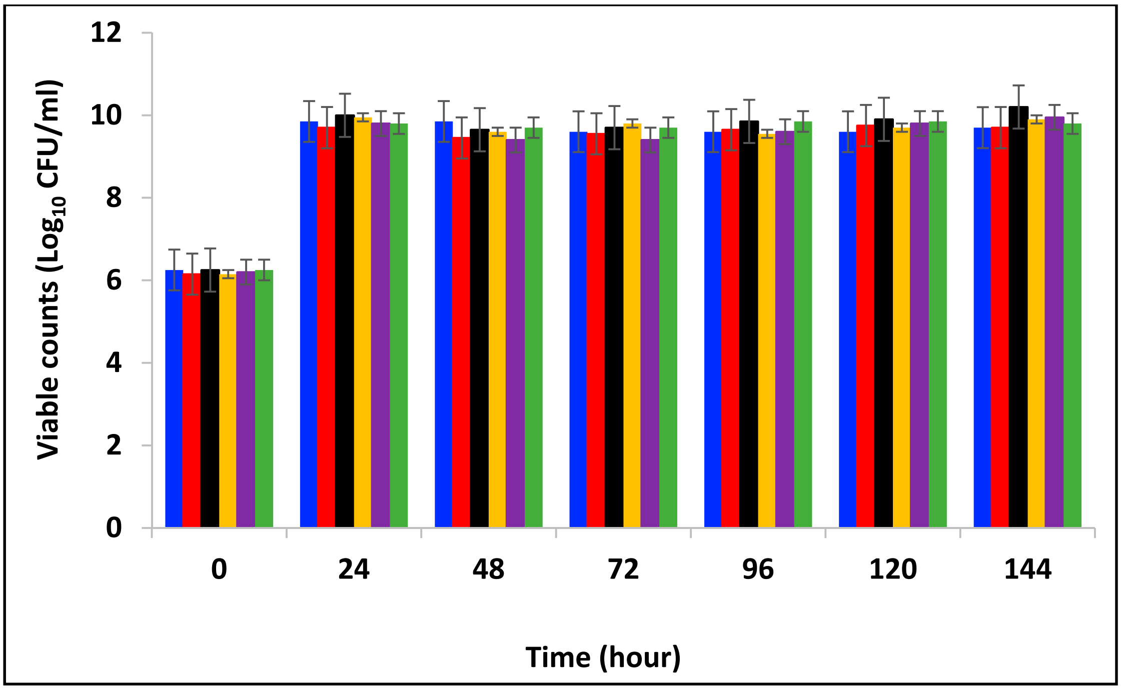

Figure S6. Growth of *L. delbrueckii* subspecies *bulgaricus* during subsequent storage at 25°C of the milk fermented at 43°C for 24 h subsequently incorporated with black pepper extract (BPE) combined with cinnamon extract (CE) at different concentrations. Concentrations are in % (w/v), values are the mean of three individual replicates (means ± SD). Control [■]; 0.5% BPE alone [■]; 0.1875% CE + 0.125% BPE [■]; 0.125% CE + 0.25% BPE [■]; 0.0625% CE + 0.375% BPE [■]; 0.25% CE alone [■].

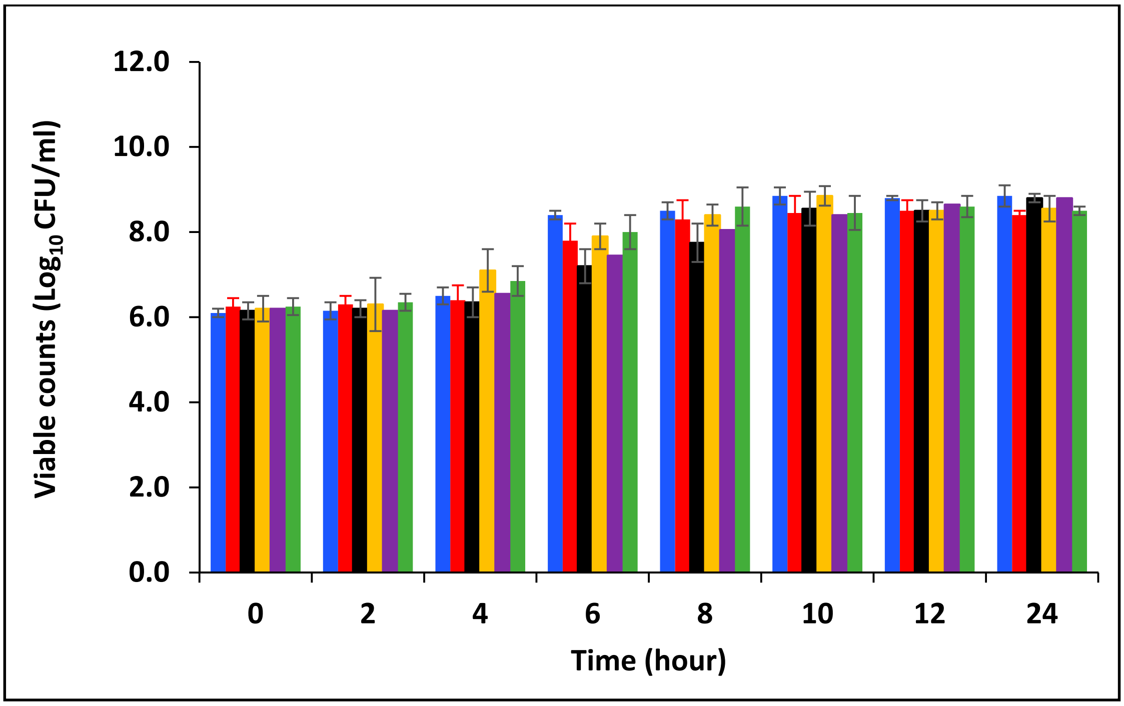

Figure S7. Growth of *S. thermophilus* in fermenting milk in the presence of essential oils of black pepper (BPE) and cinnamon extracts (CE) at different concentrations at 25°C for 24 h. Concentrations are in % (w/v), values are the mean of three individual replicates (means ± SD). Control [■]; 0.5% BPE alone [■]; 0.1875% CE + 0.125% BPE [■]; 0.125% CE + 0.25% BPE [■]; 0.0625% CE + 0.375% BPE [■]; 0.25% CE alone [■].

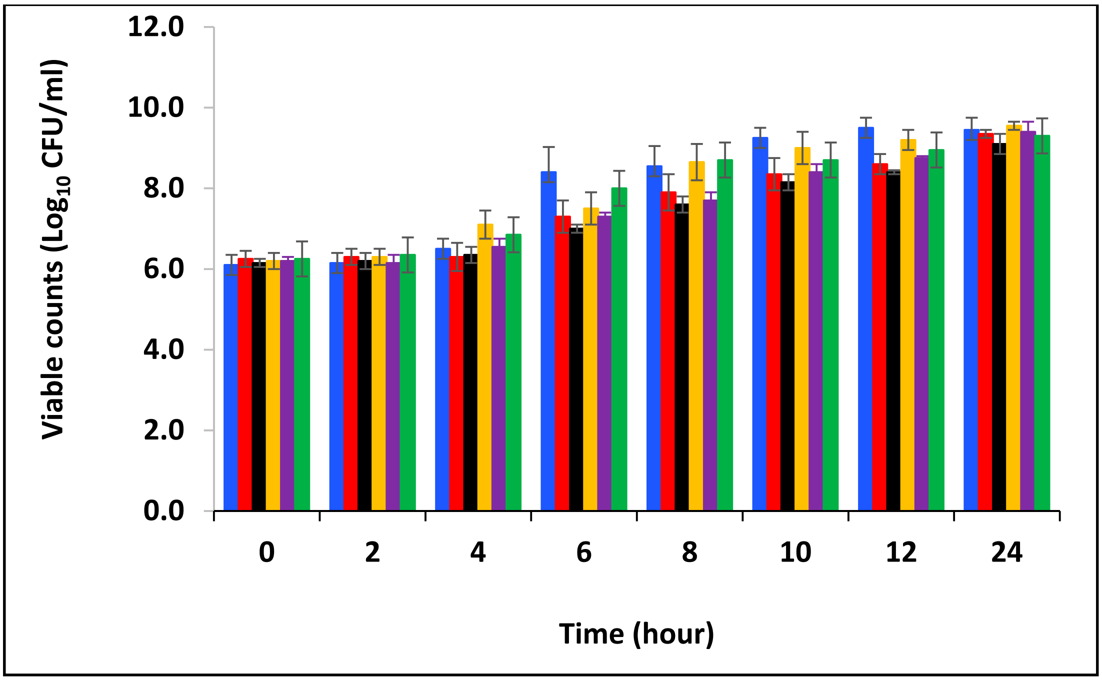

Figure S8. Growth of *S. thermophilus* in fermenting milk in the presence of essential oils of black pepper (BPE) and cinnamon extracts (CE) at different concentrations at 43°C for 24 h. Concentrations are in % (w/v), values are the mean of three individual replicates (means ± SD). Control [■]; 0.5% BPE alone [■]; 0.1875% CE + 0.125% BPE [■]; 0.125% CE + 0.25% BPE [■]; 0.0625% CE + 0.375% BPE [■]; 0.25% CE alone [■].

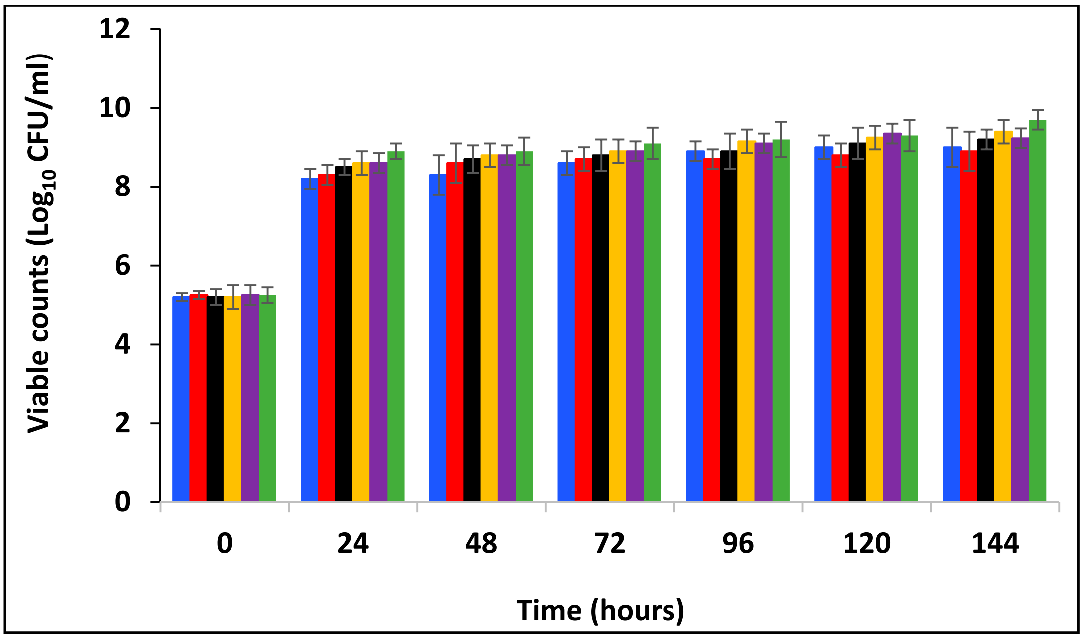

Figure S9. Growth of *S. thermophilus* in fermenting milk in the presence of essential oils of black pepper (BPE) and cinnamon extracts (CE) at different concentrations fermented at 25°C for 24 h. Concentrations are in % (w/v), values are the mean of three individual replicates (means ± SD). Control [■]; 0.5% BPE alone [■]; 0.1875% CE + 0.125% BPE [■]; 0.125% CE + 0.25% BPE [■]; 0.0625% CE + 0.375% BPE [■]; 0.25% CE alone [■].

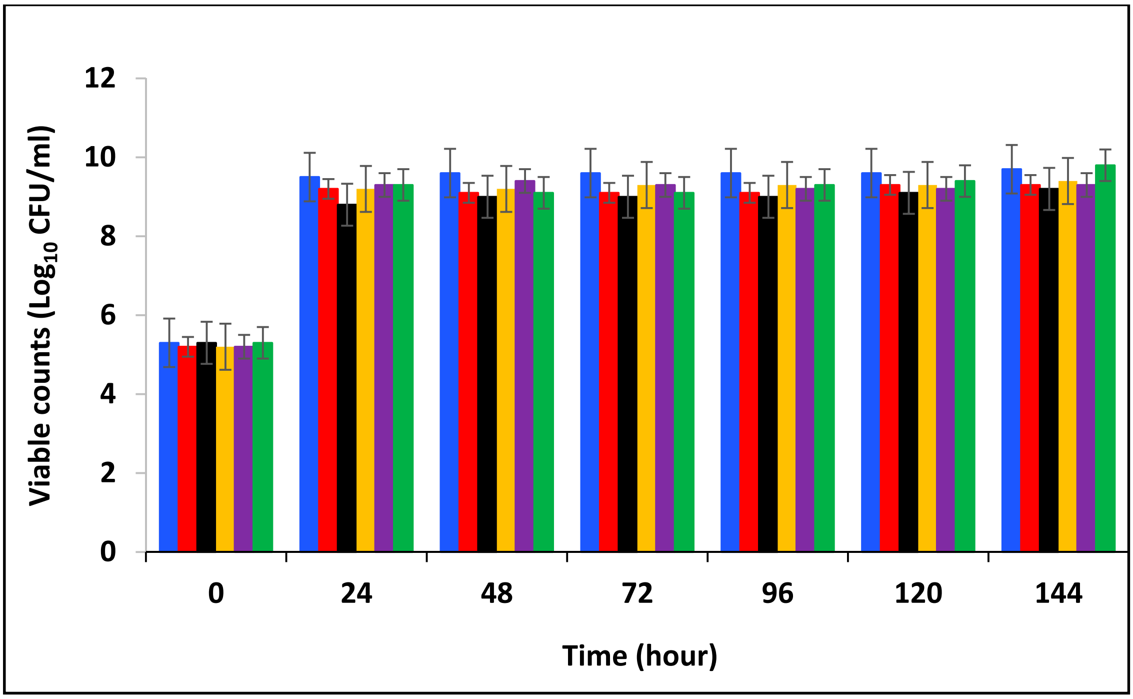

Figure S10. Growth of *S. thermophilus* in fermenting milk in the presence of essential oils of black pepper (BPE) and cinnamon extracts (CE) at different concentrations during storage at 25°C after fermentation at 43°C for 24 h. Concentrations are in % (w/v), values are the mean of three individual replicates (means ± SD). Control [■]; 0.5% BPE alone [■]; 0.1875% CE + 0.125% BPE [■]; 0.125% CE + 0.25% BPE [■]; 0.0625% CE + 0.375% BPE [■]; 0.25% CE alone [■].

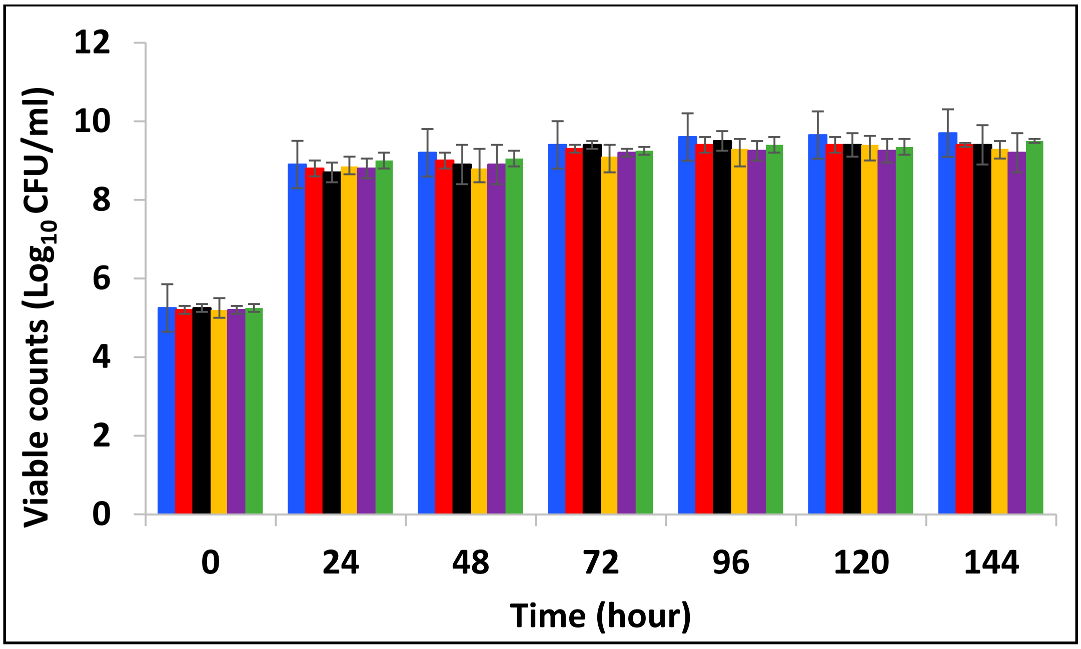

Figure S11. Survival of *S. thermophilus* during subsequent storage at 25°C of the milk fermented at 25°C for 24 h subsequently incorporated with black pepper extract (BPE) combined with cinnamon extract (CE) at different concentrations. Concentrations are in % (w/v), values are the mean of three individual replicates (means ± SD). Control [■]; 0.5% BPE alone [■]; 0.1875% CE + 0.125% BPE [■]; 0.125% CE + 0.25% BPE [■]; 0.0625% CE + 0.375% BPE [■]; 0.25% CE alone [■].

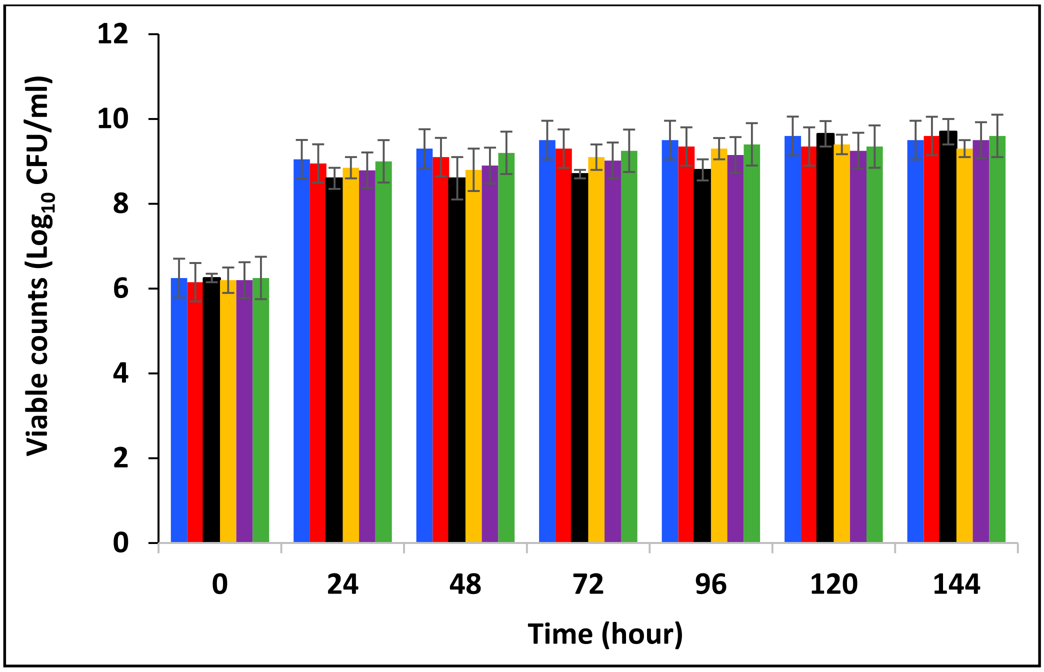

Figure S12. Survival *S. thermophilus* during subsequent storage at 25°C of the milk fermented at 43°C for 24 h then incorporated with black pepper (BPE) and cinnamon extracts (CE) at different concentrations. Concentrations are in % (w/v), the mean of three individual replicates (means ± SD). Control [■]; 0.5% BPE alone [■]; 0.1875% CE + 0.125% BPE [■]; 0.125% CE + 0.25% BPE [■]; 0.0625% CE + 0.375% BPE [■]; 0.25% CE alone [■].
